# Clinical, genetic, and molecular characterization of hyperphosphatasia with mental retardation: a case report and literature review

**DOI:** 10.1186/s13000-019-0902-5

**Published:** 2019-11-04

**Authors:** Layal Abi Farraj, Wassim Daoud Khatoun, Naji Abou Chebel, Victor Wakim, Katia Dawali, Michella Ghassibe-Sabbagh

**Affiliations:** 1 Department of Natural Sciences, School of Arts and Sciences, Lebanese American University, Beirut, Lebanon; 2 School of Medicine, Lebanese American University, Beirut, Lebanon

**Keywords:** Cleft palate, *PGAP3*, Lebanese population, Syndromic, HPMRS

## Abstract

**Background:**

Hyperphosphatasia with mental retardation syndrome (HPMRS) is a recessive disorder characterized by high blood levels of alkaline phosphatase together with typical dysmorphic signs such as cleft palate, intellectual disability, cardiac abnormalities, and developmental delay. Genes involved in the glycosylphosphatidylinositol pathway and known to be mutated in HPMRS have never been characterized in the Lebanese population.

**Case presentation:**

Herein, we describe a pair of monozygotic twins presenting with severe intellectual disability, distinct facial dysmorphism, developmental delay, and increased alkaline phosphatase level. Two individuals underwent whole exome sequencing followed by Sanger sequencing to confirm the co-segregation of the mutation in the consanguineous family. A biallelic loss of function mutation in *PGAP3* was detected. Both patients were homozygous for the c.203delC (p.C68LfsX88) mutation and the parents were carriers confirming the founder effect of the mutation. High ALP serum levels confirmed the molecular diagnosis.

**Conclusion:**

Our findings have illustrated the genomic profile of *PGAP3*-related HPMRS which is essential for targeted molecular and genetic testing. Moreover, we found previously unreported clinical findings such as hypodontia and skin hyperpigmentation. These features, together with the novel mutation expand the phenotypic and genotypic spectrum of this rare recessive disorder.

## Background

Orofacial clefts (OFCs) are the most common congenital craniofacial birth defects [10]. Their prevalence varies widely between populations with an average of 1/700 live births [10, 19]. OFCs arise as a result of genetic and environmental factors interfering abnormally with a set of coordinated events leading to the development of the craniofacial processes between the 4th and 8th gestational weeks [6]. According to genetic and epidemiological risk factors, OFCs have been divided into cleft lip with or without the palate (CL/P) and cleft palate only (CPO) [9]. Clefts are known for their complex etiology and lifelong morbidity [9]. OFCs are mainly nonsyndromic (70% of CL/P and 50% of CPO) [9]. A large number of the genetic factors involved in the remaining 30% syndromic clefts has been identified through the advancement of the genetic screening techniques [3, 8, 23]. Hyperphosphatasia with mental retardation syndrome (HPMRS) (OMIM # 239300) was first described in 1970, and was later designated as Mabry Syndrome [4, 17]. Typical facial dysmorphic signs such as cleft palate, intellectual disability, cardiac abnormalities, and brachytelephalangy are frequently described in affected individuals [1, 11, 15]. Elevated serum levels of alkaline phosphatase (ALP) have been recorded in several patients [1, 8].

Characterizing mutations underlying diseases with Mendelian patterns of inheritance by using exome sequencing is a powerful approach to explain the etiology of the disease. As part of a study of the genetic basis of different syndromic cleft phenotypes in the Lebanese population, we report the results of exome sequencing of four individuals from the same family. Our data shows novel variants in the previously established causative gene, *PGAP3* (OMIM #611801): a new phenotypic relationship with HPMRS.

## Case presentation

### Data collection

Since establishing the craniomaxillofacial research project at the Lebanese American University, we have examined over 100 patients with orofacial dysmorphic features, including a pair of twins suffering from a severe developmental syndrome. Participants provided written informed consent prior to filling out the questionnaire or giving blood samples. Parents of the minor participants provided a written declaration of consent as they were legally authorized representatives. Research and data collection were carried out in compliance with the Helsinki Declaration, with the approval of the LAU Institutional Review Board and the Committee on Human Subjects in Research (CHSR). De-identified data about the neurological, muscular, epithelial, and connective tissues related development was collected and all medical problems were obtained from medical charts.

ALP levels were measured in all patients. Information including brain imaging, EEG, skin biopsies, echocardiograms, and thyroid profile were also performed. Parents were examined and genomic DNA was extracted from peripheral blood of the patients and their two parents using the standard phenol-chloroform extraction procedure.

### Genetic analysis

Whole exome enrichment was performed on genomic DNA using the SureSelect Human All Exon V6 (Agilent). Whole exome sequencing was performed on an Illumina Hi-Seq 3000 platform using a 150 bp paired-end protocol with an average depth of 60x. Reads were aligned against the human assembly GRCh37 using Burrows-Wheeler Aligner (BWA). Data analysis was performed using SAMtools [16], Picard [5], the Genome Analysis Toolkit (GATK) [18] and Variant Effect Predictor (VEP). Polymorphisms were removed by reference to their population frequencies by searching for such mutations in genetic disease databases including OMIM (http://omim.org), HGMD (http://www.hgmd.cf.ac.uk/ac/index.php), ClinVar (https:www.ncbi.nlm.nih.gov/clinvar), the database of the 1000 Genomes Project (http://www.1000genomes.org/), ESP6500 (http://evs.gs.washington.edu/EVS/), and ExAC (http://exac.broadinstitute.org). The effects of mutations on protein function were predicted with the aid of PolyPhen 2 (http://genetics.bwh.harvard.edu/pph2/), SIFT (http://sift.jcvi.org), and CADD (https://cadd.gs.washington.edu/). The prioritization of genes was conducted using Pubmatrix (https://pubmatrix.irp.nia.nih.gov/) followed by further assessment in relation to clinical characteristics.

### Clinical characteristics

**Fig. 1:**
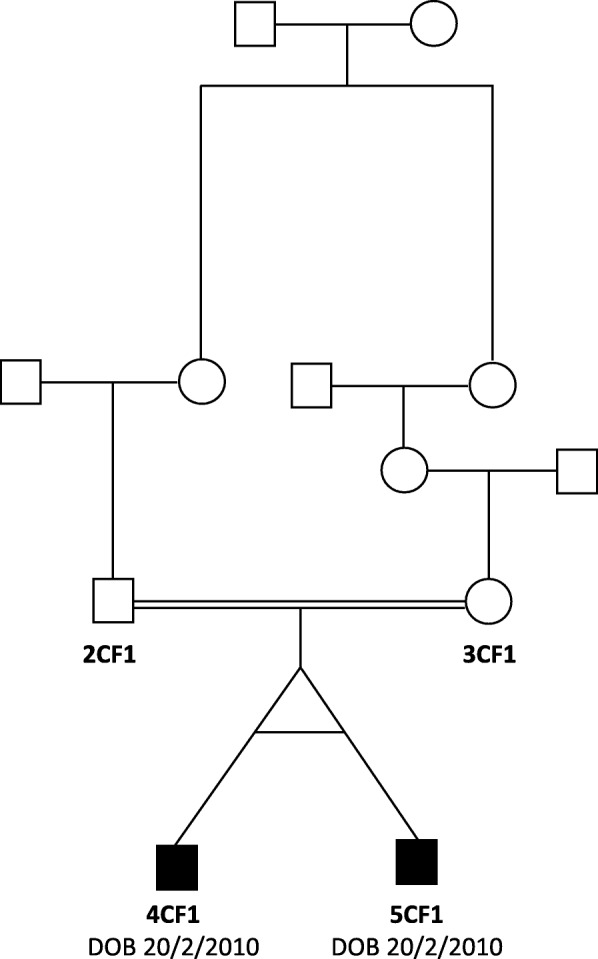
Pedigree of family. Filled symbols, affected;  , consanguinity;, monozygotic twins

There was a first-degree consanguinity between the parents of the twin pair (Fig. 1). No family history of HPMRS was recorded and both parents reported to be young and healthy at the time of conception. The twins were nine-year-old at the time of data collection. They presented hypotonia with a lack of voluntary coordination of muscle movements prohibiting them from walking, absence of speech, and abnormalities in the eye movement with down-slanting eyelids. They both suffered from growth retardation and epilepsy. No signs of autism or hyperactivity were recorded. Head magnetic resonance imaging showed evidence of 2 × 2 cm area of bleed in the left temporal lobe of both affected individuals with the presence of an abnormal vein. No arterial abnormality was seen. The pattern of gyration and myelination was normal as well as the ventricles. Skin biopsy following the description of skin hyperpigmentation confirmed the diagnosis of incontinentia pigmenti, although the WES data did not show any mutation in *IKBKG*, previously reported to be linked to the condition [25]. The twins had typical facial features with a wide nasal bridge, tent-shaped lips, and a cleft of the anterior and posterior palate together with typical recurrent ear infections. Gastroscopy in one of the twins, 4CF1, showed the presence of marked esophagitis, large hiatal hernia, petechial gastritis, and minimal nodular duodenitis. In the other twin, 5CF1, cardiac ultrasonography showed a congenital ventricular septal defect with the presence of a heart murmur. He also presented a clinodactyly of the fifth digit (Fig. 1 and Table 1).

**Table 1:** Summary of the Clinical data comparing previously reported cases of *PAPG3* with our two current cases

Clinical Data	Current case report	Previous Cases Reported (26) [1, 20, 24]
Consanguinity	2/2	16/26
Cleft Palate	2/2	17/26
Postnatal Microcephaly	2/2	8/26
Short Stature	2/2	2/26
Seizures	2/2	17/26
Coarse Facies	2/2	26/26
Developmental Delay	2/2	26/26
Speech Delay	2/2	26/26
High ALP level (relative to age)	2/2	26/26
Hyperactive & Autistic Behavior	2/2	14/26
Congenital Heart Defects	1/2	2/26
**Clinodactyly**	2/2	0/26
Small teeth	0/2	1/26
**Hypodontia**	2/2	0/26
**Hyperpigmentation(Incontinentia Pigmenti)**	2/2	0/26

In bold font are the new clinical features that were not previously reported in any case of the mutation

### Mutational analysis

The analysis of the whole exome data showed that the patient 5CF1 carried a homozygous frameshift mutation in *PGAP3*, exon 2, c.203delC (p.C68LfsX88) (Fig. 1). The father (2CF1) was heterozygous for the same mutation. Sanger sequencing was used to confirm the mutation in the two above-studied individuals, and to look for its presence in the mother (3CF1) as well as the other twin (4CF1). Results showed that both parents were carriers for the mutation, and both children were homozygous for the mutation. The presence of an A > G substitution in intron 2, 110 base pairs 3′ of the c.203delC change (genomic position 39,685,888 bp), excluded the hemizygous mutation hypothesis. Although the cytosine on the position 203 has previously been shown to be substituted, the current deletion has not been reported previously in any population. No other relevant nucleotide changes were found. In order to confirm the presence of the HPMRS syndrome, a dosage of the level of ALP was done. The results showed that the ALP level was of 390 U/L and 327 U/L for 4CF1 and 5CF1 respectively.

### Structural analysis

**Fig. 2:**
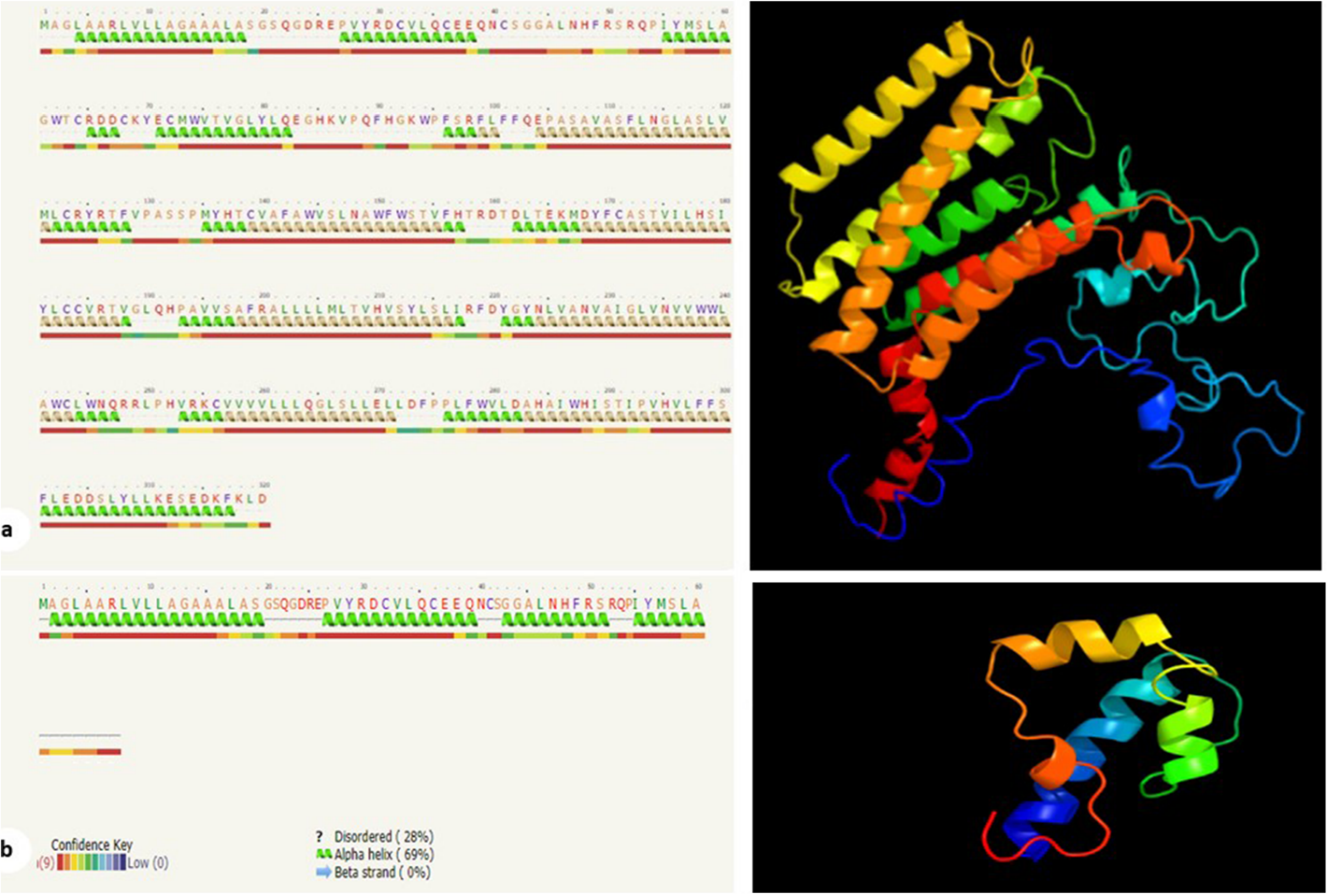
Right panel, amino acid sequence followed by secondary structure prediction. Green helices represent α-helices, and faint lines indicate coil. The line below helices indicates the confidence in the prediction, with red being high confidence and blue low confidence. The yellow helices indicate a weak prediction. Left panel, The homology modeled structure of the PGAP3 protein depicted from the Phyre2 result in cartoon format. **a**, wild-type protein; **b**, mutated protein

The mutation resulted in a stop codon replacing a cysteine residue at the 68th amino acid position. Thus, the corresponding protein has only 67 amino acids, compared to 320 amino acids in the wild type protein. Phyre2 suggested the 320 amino-acid normal protein to present seven transmembrane alpha helices spanning amino acids 99 to 296 [13]. However, since the mutated protein has only the first 67 amino acids, no transmembrane helices in this protein were suggested by Phyre2 [13]. This indicates that the protein is no longer capable of getting integrated in the membrane of Golgi apparatus, as the literature states [22]. The templates to which both the normal and the mutated proteins were aligned in the Phyre2 software further support this hypothesis (Fig. 2).

## Discussion and conclusions

In the 2001 version of the London Dysmorphology Database, 487 monogenic syndromes have been reported to be associated with ORFs [7]. As part of the effort of studying the genetic basis of Lebanese families presenting syndromic clefts, we were able to identify a novel *PGAP3*, “post-GPI attachment top 3”, mutation in one consanguineous family. The latter mutation suggested that the family presented a hyperphosphatasia with mental retardation syndrome, falling under the monogenic syndrome categorization. In order to confirm that the dysmorphic features encountered in the family are part of the HPMRS syndrome, a dosage of the level of ALP was done. The elevated levels of ALP in the blood confirmed the molecular diagnosis in the family. Interestingly, the level of ALP is 1.03 and 1.24 higher than the normal ALP range for boys between 7 and 9 years (86–315 U/L).

*PGAP3* encodes a glycosylphosphatidylinositol (GPI)-specific phospholipase that removes unsaturated fatty acids from GPI anchored protein in the Golgi apparatus where it primarily localizes and performs the first step of fatty acid remodeling [22]. The GPI-anchored proteins (GPI-APs) and lipid rafts proper association is mainly due to the remodeling of the constituent fatty acids on GPI. The symptoms caused by *PGAP3* mutations could be caused by the inability of the GPI-APs in cells to localize on lipid rafts because of their non-remodeled lipid structure, despite the presence of normal levels of PGAP3 in the cells [14].

To date, only 16 pathogenic *PGAP3* mutations have been identified in patients with HPMRS [1, 2, 12, 14, 20, 21, 24, 26]. Ten out of the 16 mutations are in exon 3 and exon 7 which are considered hotspots for mutations. The majority of these mutations are missense mutations; however, frameshift, splice site, and 3’UTR mutations were reported. The mutation reported in this case report raises the number of mutations to 17 mutations. It is a homozygous frameshift mutation located in exon 2, c.203delC (p.C68LfsX88), affecting monozygotic twins resulting from a consanguineous marriage.

The monozygotic twins have features concordant with an HPMRS phenotype although they showed the most severe spectrum of the phenotype. In addition to signs already associated with HPMRS, they also showed clinodactyly, hypodontia, as well as hyperpigmentation, a phenotype that has never been reported in HPMRS caused by a *PGAP3* mutation (Table 1).

In summary, we reported a novel homozygous mutation in *PGAP3* in a consanguineous Lebanese cleft syndromic family associated with hyperphosphatasia with mental retardation syndrome. Novel features not reported as part of this syndrome before were present. This case report shows that ORFs associated with global developmental delay and facial dysmorphism with elevated alkaline phosphatase are important clues for HPMRS. In addition, it shows that whole exome sequencing helps increase the diagnostic yield for heterogeneous conditions such as syndromic ORFs encountered more commonly in consanguineous families. Molecular diagnosis might enable a proper recognition of the associated syndromes and anomalies with ORFs by finding the causative agents, which is essential for informative genetic counselling, prenatal diagnosis, targeted treatment and prevention in case it is possible.

## Data Availability

The datasets generated and/or analyzed in this case report are available from the corresponding author upon reasonable request.

## Abbreviations

ALP: Alkaline phosphatase
CHSR: Committee on Human Subjects in Research
CPO: Cleft palate only
GPI: Glycosylphosphatidylinositol
GPI-Aps: GPI-anchored proteins
HPMRS: Hyperphosphatasia with mental retardation syndrome
OFCs: Orofacial clefts
WES: Whole Exome Sequencing
